# High Structural Stability, High Compressive Strength, Excellent Thermal Insulation and Mechanism of Needled Quartz Fiber Felt/Phenolic Aerogel Composites

**DOI:** 10.3390/polym18060705

**Published:** 2026-03-13

**Authors:** Dongmei Zhao, Kaizhen Wan, Xiaobo Wan, Yiming Liu, Jian Li, Minxian Shi

**Affiliations:** 1 School of Materials Science and Engineering, Wuhan University of Technology, Wuhan 430070, China; 2 Hainan Institute, Wuhan University of Technology, Sanya 572000, China; 3 School of Automotive Materials, Hubei University of Automotive Technology, Shiyan 442002, China

**Keywords:** phenolic aerogel, needled quartz fiber felt, compression behavior, thermal insulation, thermal conductivity

## Abstract

A lightweight composite that simultaneously exhibits high strength and excellent thermal insulation is of great interest for thermal protection applications. In this study, dimensionally stable needled quartz fiber felt-reinforced phenolic aerogel composites were prepared using vacuum impregnation, sol–gel, and ambient pressure drying. The composites exhibit a multiscale porous structure formed by interconnected nanometer polymer skeletons and micronscale fibers. By regulating the thermoplastic phenolic resin concentration in the precursor solution, the pore structure of the material was refined; the average particle diameter reduced from 99.76 nm to 38.91 nm, and the average pore diameter decreased from 216.79 nm to 49.53 nm. At a phenolic resin concentration of 25%, the composite exhibits outstanding thermal insulation and mechanical properties: a low thermal conductivity of 0.0646 W·m^−1^·K^−1^ at room temperature, with a mere 19.5 °C temperature rise on the sample backside after 1800 s heating at 200 °C, and compressive strengths of 7.70 MPa in the XY-direction and 3.87 MPa in the Z-direction (at 10% strain). X-ray micro-CT characterized the internal structural evolution during loading, revealing a failure mechanism dominated by fiber buckling. Theoretical models and experimental data were used to analyze and quantify the contribution rates of gas and solid heat conduction in NQF/PR aerogel composites, with solid conduction accounting for over 80%. Combined with microstructural evolution, the mechanism for the high thermal insulation efficiency of NQF/PR aerogel composites was elucidated. This study prepared NQF/PR aerogel composites with promising application potential. By systematically evaluating their compressive behavior and quantifying the respective contributions of gas and solid conduction, this work provides a methodological framework to guide the rational design of similar aerogel composites.

## 1. Introduction

With the continuous development of aerospace engineering, aircrafts face increasingly severe aerothermal environments [1,2]. The thermal environment is characterized by high heat flux, substantial total heating, and strong mechanical stress induced by aerodynamic shocks and thermal loads. This imposes extremely high demands on the thermal protection system of the aircraft. As the core functional component of the thermal protection system, the performance of the thermal protection materials is crucial for ensuring the structural integrity of the aircraft in the extreme aerodynamic thermal environment and is directly related to the success or failure of the flight mission [3]. Therefore, thermal protection materials need to possess lightweight properties, highly efficient thermal insulation capabilities, and excellent mechanical performance.

In recent years, phenolic aerogels have attracted extensive research attention due to their outstanding properties such as low density, ablation resistance, high carbon residue rate, and low thermal conductivity [4,5,6]. However, issues like high brittleness, insufficient mechanical properties, large volumetric shrinkage, and easy crushing significantly limit their application scope. Currently, these challenges are commonly addressed through the following two approaches [7]. One approach involves introducing crosslinking agents to reinforce the network structure of aerogel, thereby enhancing its mechanical strength [8]. He et al. [9] employed cobalt acetate as a crosslinking agent and used freeze-drying to prepare a resorcinol–formaldehyde aerogel with a cobalt-coordinated crosslinked skeleton. The appropriate amounts of cobalt acetate enabled the aerogel to form a thick pearl necklace-like skeleton structure. However, at relatively high Co^2+^ levels, stable network structures proved difficult to form. Yang et al. [10] reported a synthetic route for polyurethane-modified phenolic resin aerogels. Although structural modifications improved the mechanical properties, reliance on highly efficient crosslinking agents incurs high costs and involves complex chemical reactions with lengthy processes, substantially increasing economic and time costs. Therefore, the introduction of crosslinking agents is not recommended for those pursuing simple, efficient preparation strategies.

Another method employs high-porosity preform materials for reinforcement, such as foams [11], 2.5D woven fabrics [12], 3D woven fabrics, and needled fiber felts [13]. Wu et al. [14] reinforced phenolic aerogel with melamine foam, and the resulting composite exhibited a compressive strength and modulus of only 16.19 MPa and 24.10 MPa at 70% strain, respectively. These values remained low, indicating a need for more efficient reinforcement strategies. Niu et al. [15] enhanced the mechanical properties of phenolic aerogel by using 2.5D quartz fabric. However, the introduction of this reinforcement structure significantly increased the material density to 1.32 g/cm^3^ and raised the thermal conductivity to 0.21 W·m^−1^·K^−1^. Jiang et al. [16] reinforced phenolic aerogel with integrally woven carbon fiber preform, resulting in an ablation layer density of 0.97 g/cm^3^ and a thermal conductivity as high as 0.309 W·m^−1^·K^−1^. High-density fibers such as 2.5D and 3D woven fabrics are introduced into phenolic aerogels at the expense of their light weight and thermal insulation properties.

Needled fiber felt is a three-dimensional fabric produced by mechanically intertwining and stacking fiber cloth and fiber felt through a needle-punching process. It exhibits certain mechanical strength and has a lower density than 2.5D and 3D woven fabrics. Compared to foams or woven fabrics, needled fiber felts as a reinforcement enable aerogel composites to achieve a superior balance of thermal insulation and mechanical strength. The introduction of fiber felt also inhibits volumetric shrinkage during the drying process of wet gels. Zhao et al. [17] introduced quartz fiber felt into aerogel, significantly reducing volumetric shrinkage from 24.8–32.7% in pure PRAs to 4.5–7.6%. In addition, needled fiber felt also has the advantages of a simple preparation process, low cost and high flexibility [18].

Common needled fiber felts include carbon fiber felt and quartz fiber felt. Carbon fiber felt, due to its inherently high thermal conductivity, introduces complex effects to the thermal insulation properties of the material. Liu et al. [19] prepared phenolic impregnated carbon ablation material reinforced with carbon fiber felt, exhibiting a thermal conductivity of 0.113 W·m^−1^·K^−1^ at room temperature. Cheng et al. [20] reported a lightweight carbon/phenolic composite with a thermal conductivity of 0.093 W·m^−1^·K^−1^ at room temperature.

In comparison, quartz fiber felt exhibits a more pronounced overall advantage in thermal insulation while maintaining reinforcement effects due to its lower thermal conductivity. Wang et al. [21] prepared gradient quartz fiber-reinforced phenolic aerogel composites with surface densification, achieving a rebound compressive strength of 0.48 MPa in the lightweight inner layer and a thermal conductivity below 0.03 W·m^−1^·K^−1^. Liu et al. [22] investigated the influence of quartz fiber characteristics on the structure and properties of aerogel composites, finding that reducing the quartz fiber diameter suppresses radiative heat transfer, thereby lowering the thermal conductivity. Overall, quartz fiber felt demonstrates significant potential in imparting exceptional thermal insulation and mechanical properties to aerogels. However, the microstructural failure behavior and thermal insulation mechanisms of phenolic aerogel composites reinforced with quartz fiber felt require further investigation.

To address the issues mentioned above, this study prepared quartz fiber-reinforced phenolic resin aerogel composites (NQF/PR) using a combined method of vacuum impregnation, sol–gel reaction, and ambient pressure drying. By adjusting the phenolic resin concentration in the aerogel precursor solution, NQF/PR aerogel composites with different porosities were prepared. The internal structural failure behavior of the material under compressive loading was revealed using X-ray micro-CT. Through a combination of experiment and theory, the contributions of gas and solid conductivity to the overall thermal conductivity were quantitatively analyzed, elucidating the mechanism behind the high-efficiency thermal insulation of NQF/PR aerogel composites.

## 2. Experimental Section

### 2.1. Materials

Thermoplastic phenolic resin (TPPR, 2123-3) was supplied by Henan Zhongfan Dongsheng New Materials Technology Co., Ltd., Xinxiang, China, with a true density of 1.275 ± 0.025 g/cm^3^ after curing. The needled quartz fiber felt (NQF, SJ105-2000, SiO_2_ content ≥ 99.9%) was obtained from Henan Shenjiu Tianhang New Materials Co., Ltd., Zhengzhou, China, with an apparent density of 0.2 g/cm^3^ and a true density of 2.2 g/cm^3^. P-toluenesulfonic acid (TsOH, C_7_H_8_O_3_S, 65% in H_2_O), n-butanol (C_4_H_10_O, 99.5%), and deionized water were supplied by Shanghai Macklin Biochemical Co., Ltd. (Shanghai, China). Hexamethylenetetramine (HMTA, ≥98.0%) was provided by Sinopharm Chemical Reagent Co., Ltd. (Shanghai, China). All chemicals were used as received.

### 2.2. Preparation of NQF/PR Aerogel Composites

**Figure 1:**
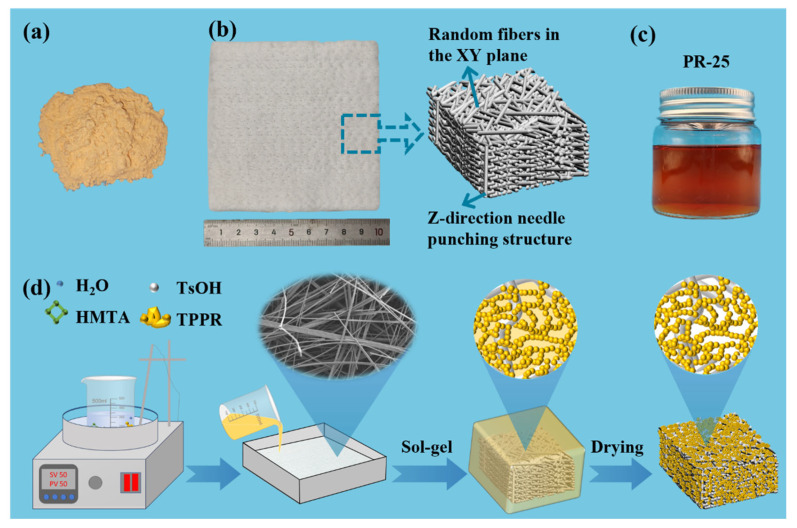
Raw materials and preparation process. (**a**) Thermoplastic phenolic resin. (**b**) Needled quartz fiber felt. (**c**) PR-25 phenolic aerogel precursor solution. (**d**) Schematic illustration of NQF/PR aerogel composite preparation.

NQF/PR aerogel composites were prepared by impregnating needled quartz fiber felt with a phenolic aerogel precursor solution, followed by sol–gel processing and ambient pressure drying. Figure 1a,b shows the phenolic resin and needled quartz fiber felt used in this study. The preparation process of NQF/PR is illustrated in Figure 1d. Firstly, H_2_O, HMTA, TsOH and TPPR were dissolved in n-butanol in a 50 °C water bath (KER-JS, Wuhan Keer Instrument Equipment Co., Ltd., Wuhan, China) to prepare five different concentrations (phenolic resin mass fraction) of aerogel precursor solutions for later use. The detailed formulations are listed in Table 1. The prepared precursor solutions were named PR-X based on their phenolic resin concentration, where X is 10, 15, 20, 25, 30. The precursor solution with a phenolic resin concentration of 25% is shown in Figure 1c. The prepared phenolic aerogel precursor solution was poured into a mold, ensuring complete impregnation of the pre-cut needled quartz fiber felt. Then, the air bubbles in the precursor solution were removed by vacuuming (DZF-6030A, Shanghai Yiheng Technology Instrument Co., Ltd., Shanghai, China) for 15 min at room temperature to ensure the fiber felt was completely impregnated with the precursor solution. Subsequently, the mold was sealed and transferred to an 80 °C oven (ADX-DGG-9246A, Wuhan Andexin Testing Equipment Co., Ltd., Wuhan, China) for sol–gel reaction. The obtained wet gel composites were dried under ambient pressure (65 °C for 12 h, 85 °C for 8 h, 100 °C for 4 h, 120 °C for 4 h) to produce the NQF/PR aerogel composites. According to the phenolic resin concentration in the precursor solution, the prepared composites were designated as NQF/PR-X, where X is 10, 15, 20, 25, 30.

### 2.3. Characterization Methods

The microstructure of the NQF/PR aerogel composites was characterized using scanning electron microscope (SEM, ZEISS Gemini 300, Oberkochen, Germany). The compressive behaviors were evaluated on samples (10 mm × 10 mm × 10 mm) using an electronic universal testing machine (RGM-2100, Shenzhen Rigel Company, Shenzhen, China) at a loading rate of 1 mm/min. Five samples were tested for each type. The thermal conductivity of materials was determined using a thermal constant analyzer (TPS 2500S, Hot Disk AB, Gothenburg, Sweden). The sample dimensions were 20 mm (diameter) × 10 mm (height), with three samples tested for each type. The thermal stability of the samples in air was characterized using a comprehensive thermal analyzer (STA449F3, NETZSCH Group, Selb, Germany) over a temperature range from room temperature to 1200 °C at a heating rate of 10 °C/min. To evaluate the thermal insulation performance of the material in practical applications, a heating plate (BY1010, Dongguan Bangyuan Electronics Co., Ltd., Dongguan, China) was used to simulate a heating environment. Samples measuring 50 mm × 50 mm × 20 mm were heated at a constant temperature of 200 °C. An infrared thermal imager (Hikvision H21 Pro+, Hangzhou Microimage Software Co., Ltd., Hangzhou, China) was employed to record the temperature on the backside of the samples. To assess the thermal insulation performance of the sample at high temperatures, a 100 mm × 100 mm × 10 mm sample was exposed to a 1300 °C butane flame at a distance of approximately 10 cm for 40 min. The surface temperature on the backside was recorded using an infrared thermal imager (Hikvision H21 Pro+). The apparent density of the material was calculated using Equation (1), based on the sample external dimensions and mass measured with a vernier caliper and electronic balance, respectively. The porosity of the material was calculated using Equation (2). The gel particle diameter and pore diameter distributions were analyzed based on SEM images using nano measurement software (Nano Measurer 1.2). The 3D structure of the samples was scanned using micro-CT (Xlab-160, Suzhou Wkuang Technology Co., Ltd., Suzhou, China), and the internal morphology was characterized based on the reconstructed images. Nitrogen adsorption–desorption isotherms of the NQF/PR were measured at 77 K using a fully automated specific surface area and porosity analyzer (ASAP 2460, Micromeritics, Norcross, GA, USA). Before testing, the samples were degassed at 120 °C for 6 h. The specific surface area was calculated using the Brunauer–Emmett–Teller (BET) theory. The total pore volume was determined at a relative pressure of 0.99. The micropore volume and micropore surface area were calculated using the t-plot theory. The mesopore and micropore pore diameter distributions were analyzed using the BJH (Barrett–Joyner–Halenda) theory and the Horvath–Kawazoe method.

(1) ρ=m/V

where *ρ* is the apparent density, *m* is the mass, and *V* is the volume of the sample.

(2) $$\varphi =1-\rho \sum_{i=1}^{n}\frac{c_{i}}{\rho_{i}}$$

where *φ* is porosity; *c_i_* is the mass fraction of component *i*; *ρ_i_* is the true density of component *i*; *ρ* is the apparent density of the sample.

## 3. Results and Discussion

### 3.1. Macroscopic Morphology and Microstructure

**Figure 2:**
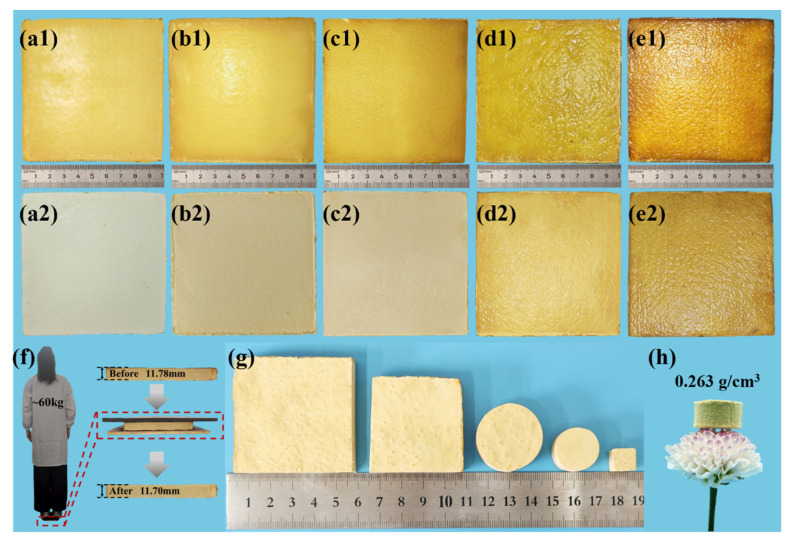
(**a1**–**e1**,**a2**–**e2**) show macroscopic images of NQF/PR-10, -15, -20, -25, and -30 before and after drying, respectively. (**f**) Digital images of NQF/PR-25 supporting a 60 kg weight of human. (**g**) Digital images of NQF/PR processed into various square and circular shapes. (**h**) NQF/PR-10 placed on a flower.

Figure 2(a1,b1,c1,d1,e1,a2,b2,c2,d2,e2) shows the macroscopic morphology of the NQF/PR aerogel composites before and after drying, respectively. Table 2 lists the dimensions of the NQF/PR before and after drying. Comparison revealed that the composite dimensions remained almost unchanged after drying. The color gradually deepened from light yellow to yellowish-brown as the phenolic resin concentration in the aerogel precursor solution increased. The overall morphology remained intact, indicating that the prepared NQF/PR composites exhibit excellent structural and dimensional stability. Figure 2f demonstrates that the NQF/PR-25 maintains structural integrity without obvious cracks after being stepped on by an adult weighing 60 kg, indicating an excellent load-bearing capacity. The property contributes to good machinability, allowing the material to be processed into specialized shapes via subtractive manufacturing, as shown in Figure 2g. This property enables the composites to effectively meet the diverse shape and size requirements in industrial production.

The physical property parameters of different samples are shown in Table 3. The concentration of phenolic resin in the aerogel precursor solution significantly affects the density and porosity of the aerogel composites. As the phenolic resin concentration gradually increased from 10% to 30%, the density of the NQF/PR rose from 0.263 g/cm^3^ to 0.484 g/cm^3^, while the corresponding porosity decreased from 84.94% to 67.69%. Higher concentrations of phenolic resin in the precursor solution result in the formation of more numerous and denser primary nanoparticles during gelation. Consequently, the crosslinked phenolic aerogel framework becomes denser, leading to increased material density and reduced porosity. As shown in Figure 2h, NQF/PR can be easily placed on flower petals.

**Figure 3:**
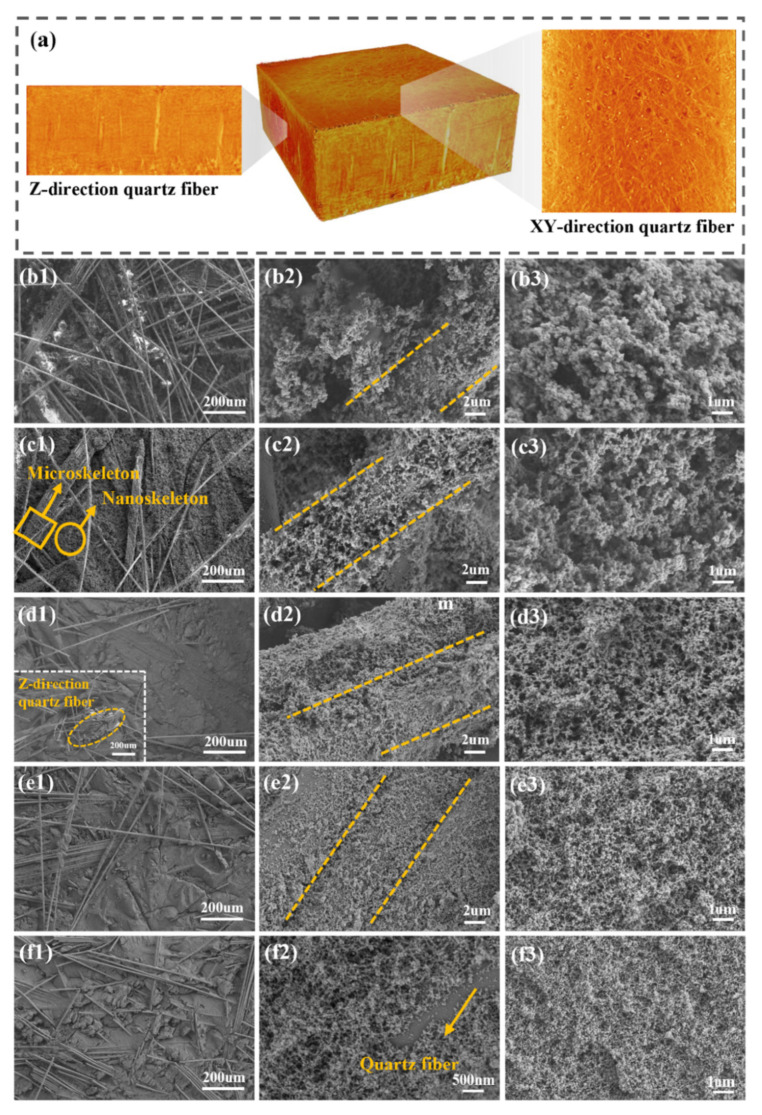
Microstructural morphology of NQF/PR. (**a**) Reconstructed 3D-CT images of NQF/PR-25. (**b1**–**b3**) NQF/PR-10. (**c1**–**c3**) NQF/PR-15. (**d1**–**d3**) NQF/PR-20. (**e1**–**e3**) NQF/PR-25. (**f1**–**f3**) NQF/PR-30.

Figure 3 displays the microstructure of the NQF/PR aerogel composites, revealing a multiscale porous structure composed of interconnected nanometer polymer skeletons and micronscale fibers. Within this structure, quartz fibers from NQF are randomly distributed within the felt layer (XY plane), forming a disordered two-dimensional network. A small number of needled fibers (Z-direction fibers) intersect these layers at an angle nearly perpendicular to the stacked plane (Figure 3a). The phenolic aerogel nanoscale skeleton fills the interstitial spaces between quartz fibers. As shown in Figure 3(b1), the NQF/PR-10 sample exhibits a relatively loose phenolic aerogel matrix due to the lower phenolic resin concentration in the precursor solution. In contrast, samples NQF/PR-15 to NQF/PR-30 (Figure 3(c1,d1,e1,f1)) demonstrate progressively denser phenolic aerogel matrices as the resin content increases.

Figure 3(b2,c2,d2,e2,f2) shows high-magnification images of the interface between the phenolic aerogel matrix and fibers. It can be observed that the aerogel uniformly attaches to the quartz fiber surface, forming an effective encapsulation. This close contact between the fibers and the aerogel matrix facilitates stress transfer. This structural foundation supports the potential of NQF/PR aerogel composites in multifunctional applications requiring thermal insulation and load-bearing capacity.

**Figure 4:**
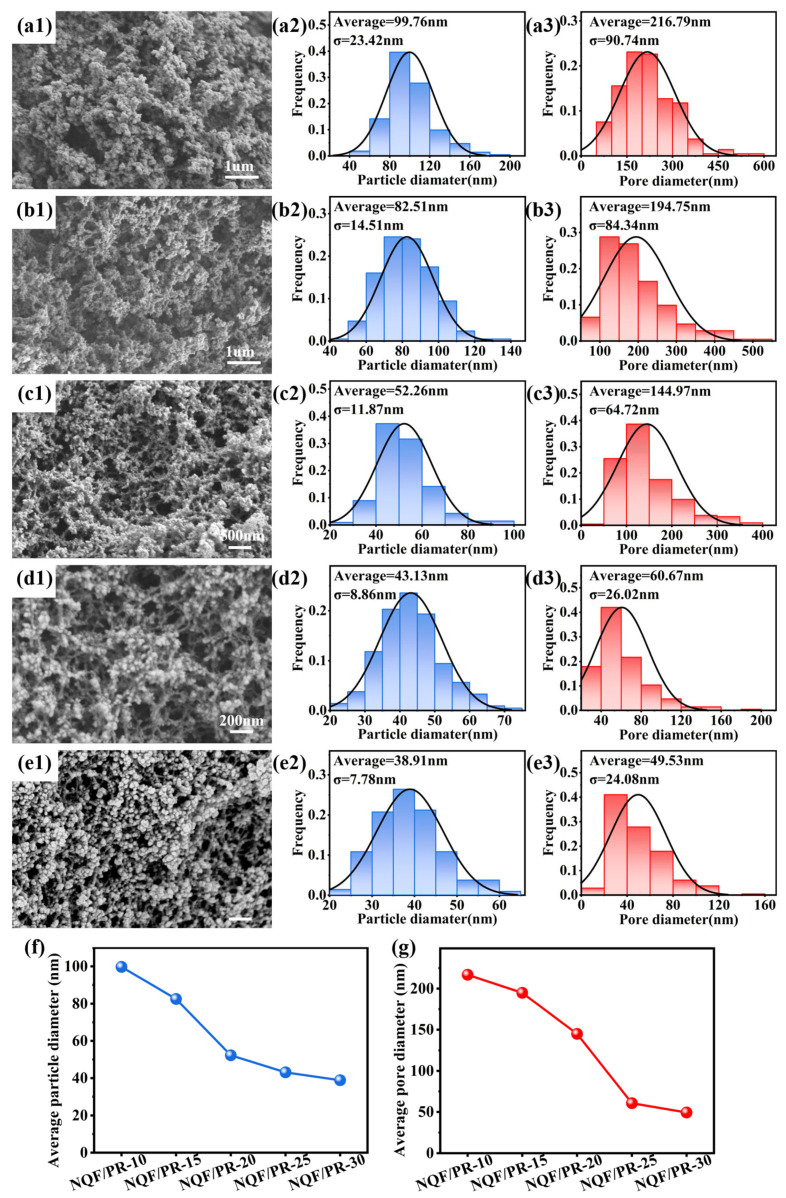
Particle and pore diameter distributions of NQF/PR. (**a1**–**a3**) NQF/PR-10. (**b1**–**b3**) NQF/PR-15. (**c1**–**c3**) NQF/PR-20. (**d1**–**d3**) NQF/PR-25. (**e1**–**e3**) NQF/PR-30. (**f**) Average particle diameter of NQF/PR. (**g**) Average pore diameter of NQF/PR.

Furthermore, high-magnification scanning images of the aerogel matrix (Figure 3(b3,c3,d3,e3,f3)) reveal that the aerogel matrix maintains a typical nanoporous bicontinuous structure, consisting of a continuous three-dimensional network formed by interconnected gel skeletons. With the gradually increasing phenolic resin content, both the aerogel particle size and pore volume exhibit a decreasing trend. To systematically quantify structural evolution, nano measurement software was employed to statistically analyze the distribution of gel particle and pore diameters, with the results presented in Figure 4(a2,b2,c2,d2,e2,a3,b3,c3,d3,e3). For direct comparison, the average particle and pore diameters of all samples were summarized in Figure 4f,g. The statistical results indicate that as the phenolic resin concentration in the aerogel precursor solution gradually increased from 10% to 15%, 20%, 25%, and 30%, the average aerogel particle diameter decreased sequentially from 99.76 nm to 82.51 nm, 52.26 nm, 43.13 nm, and 38.91 nm. Correspondingly, the average pore diameter decreased from 216.79 nm to 194.75 nm, 144.97 nm, 60.67 nm, and finally to 49.53 nm.

Compared with the existing studies, the average particle diameter and pore diameter of the aerogel composites prepared in this study were within the typical range for similar materials, while exhibiting certain advantages in structural refinement. Cheng et al. [20] reported similar aerogel composites with average particle diameters ranging from 79.53 to 124.46 nm. Sha et al. [23] achieved average aerogel particle diameters of approximately 65–100 nm and average pore diameters of 0.25–0.91 μm by adjusting the curing agent concentration. In this study, increasing the phenolic resin concentration to 30% reduced the average particle diameter to 38.91 nm and the average pore diameter to 49.53 nm. This demonstrates that controlling the phenolic resin concentration in the precursor solution enables the preparation of aerogels with smaller structural units. Higher resin concentrations generate more chemical reaction centers during sol–gel conversion, resulting in denser crosslinked network structures. This yields smaller gel particles and narrower pore structures after drying [14]. These results demonstrate that adjusting the resin concentration in the precursor solution effectively controls the aerogel nanostructure, providing a basis for further optimization of mechanical, thermal, and other physical properties.

### 3.2. Pore Structure

**Figure 5:**
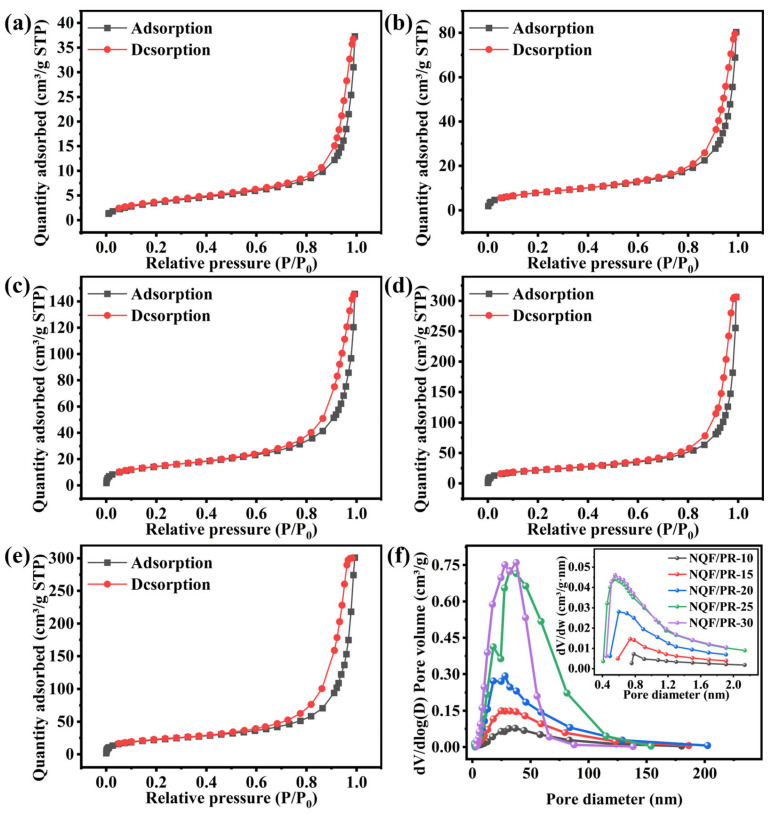
(**a**–**e**) N_2_ adsorption and desorption isotherms of NQF/PR-10, -15, -20, -25, -30 at 77 K. (**f**) BJH pore diameter distribution curves of NQF/PR, with the inset showing the Horvath–Kawazoe microporous pore diameter distribution curves.

To further analyze the pore diameter characteristics of NQF/PR aerogel composites, N_2_ adsorption tests were conducted on samples NQF/PR-10 to NQF/PR-30, with the results shown in Figure 5a–e. According to the classification criteria of the International Union of Pure and Applied Chemistry (IUPAC), both the adsorption–desorption isotherms and hysteresis loops of the NQF/PR exhibited typical Type IV isotherms and H3-type hysteresis loops, indicating the presence of a hierarchical pore structure within the material [24]. Specifically, N_2_ adsorption was observable at lower relative pressures (P/P_0_ < 0.1), indicating the presence of micropores within the material. As relative pressure increases, adsorption capacity rose gradually, primarily corresponding to multilayer adsorption of nitrogen molecules on various pore surfaces. In the high-pressure region (P/P_0_ > 0.8), adsorption exhibited a steep rise, attributed to capillary condensation effects within mesopores. The separation of adsorption and desorption isotherms at high pressure, forming the H3 hysteresis loop, further confirmed the presence of an irregular three-dimensional mesoporous network within the NQF/PR materials.

The specific surface area and pore volume of the NQF/PR were calculated based on N_2_ adsorption and desorption isotherms, with the results shown in Table 4. The NQF/PR-10 exhibited a specific surface area of 13.521 m^2^/g, with micropore surface area and volume being negligible. The low specific surface area primarily resulted from the dominant macroporous structure of NQF/PR-10, which contributed minimally to the surface area.

As the phenolic resin concentration was gradually increased from 10% to 25%, the specific surface area and total pore volume of NQF/PR increased sharply. Specifically, the specific surface area rose from 13.521 m^2^/g to 78.690 m^2^/g, an increase of nearly 6-fold, while the total pore volume increased from 0.058 cm^3^/g to 0.474 cm^3^/g, an approximately 8-fold increase. Concurrently, the surface area and volume of micropores in each sample also exhibited growth. This increase is primarily because as the concentration of phenolic resin increases, the number of chemical reaction active sites grows, leading to refined aerogel particle sizes and tighter packing. The large pores originally present between aggregates, which were difficult to detect using nitrogen adsorption, are gradually filled by gel particles. Thus, more micropores and mesoporous structures are formed, significantly enhancing the specific surface area and pore volume of the material.

When the phenolic resin concentration increased from 25% to 30%, the specific surface area gradually rose from 78.690 m^2^/g to 82.311 m^2^/g, while the total pore volume slightly decreased. Once the aerogel structure reached a certain degree of compactness, further increasing the phenolic resin concentration, it caused the gel particles to pack more densely. As a result, some existing micropores and mesopores were converted into smaller pores due to particle filling, or even gradually closing. Consequently, the specific surface area increased slowly, and the total pore volume decreased slightly. This phenomenon also indicates that increasing the phenolic resin concentration cannot indefinitely optimize the pore structure. Figure 5f shows the pore diameter distribution of the NQF/PR, indicating that mesopores are primarily in the range of 25–45 nm, and micropores are centered between 0.5 and 0.8 nm.

### 3.3. Compression Behavior and Mechanism

**Figure 6:**
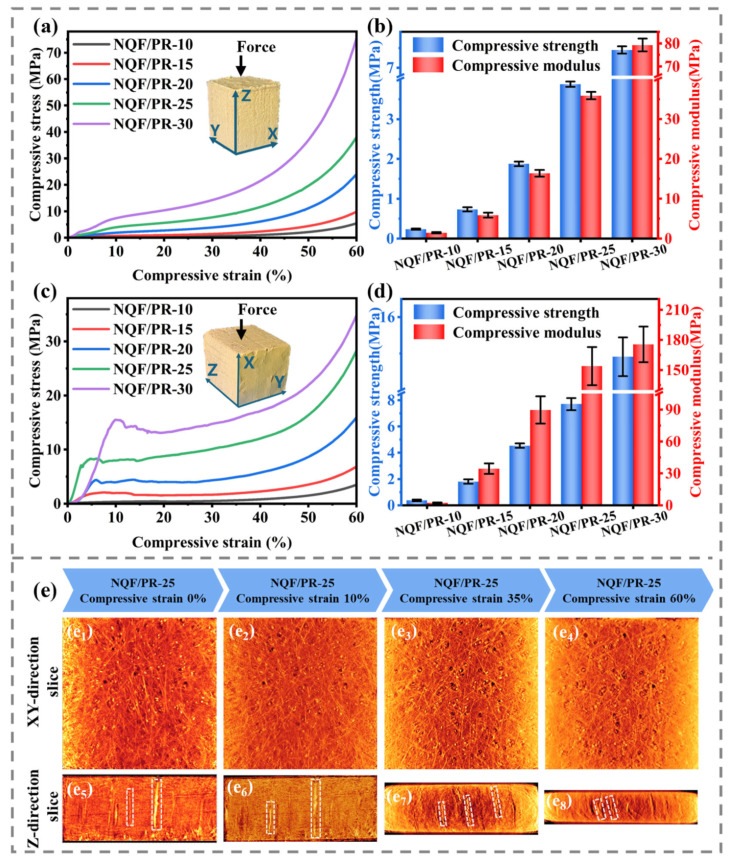
Mechanical properties of NQF/PR. (**a**,**c**) Compressive stress–strain curves in the Z- and XY-direction. (**b**,**d**) Compressive strength and modulus in the Z- and XY-direction. (**e**) CT slices under Z-direction compression. (**e_1_**–**e_8_**) represent slices in the XY and Z directions at 0%, 10%, 35%, and 60% compressive strain, respectively.

Ordinary phenolic aerogels exhibit brittle characteristics and are prone to fracture under compression. To address the problem, fiber reinforcement has been shown to be an effective strategy for improving mechanical strength [25]. Here, the compressive properties of NQF/PR aerogel composites in the Z- and XY-directions were tested using an electronic universal testing machine. Figure 6a,c displays typical compressive stress–strain curves under Z- and XY-directional loads at room temperature, respectively. These curves show a nonlinear deformation response, characteristic of porous materials, in both directions. At the final stage of compression, the stress in both directions increases sharply.

Figure 6a shows that in the Z-direction, the stress–strain curves of the aerogel composites exhibit smooth and continuous characteristics without any stress fluctuations, indicating that the samples deform continuously during compression rather than undergoing brittle fracture. The stress–strain curve in the Z-direction can be divided into three stages [26,27]. Using NQF/PR-25 as an example, the internal structural changes in the material in each stage are shown in Figure 6e. In the initial elastic stage (0–10% strain), stress increases nearly linearly with strain. During this stage, deformation primarily results from elastic compression of the aerogel skeleton and slight bending of the Z-direction fibers. Compared to the initial state, CT slices at 10% (Figure 6(e_6_)) strain reveal no significant deformation of the Z-direction fibers, nor any apparent interface failure between the aerogel matrix and fibers. Structural damage remains negligible at this stage, consistent with macroscopic linear elastic behavior.

In the yield stage, the curve slope decreases, indicating a reduced stress increase rate. This behavior is primarily attributed to irreversible compression of the phenolic aerogel matrix and buckling instability of the quartz fibers. The corresponding CT slices at 35% (Figure 6(e_7_)) strain exhibit noticeable compression and buckling of the Z-direction fibers, along with debonding between the fibers and the aerogel matrix, indicating damaged interfacial bonding. The progressive accumulation of interfacial damage corresponds to the yield stage with reduced slope in the macroscopic curve. This observation directly confirms that yielding behavior is not caused by a single factor, but rather results from the synergistic interaction between fiber buckling and interfacial debonding.

Upon reaching a certain compression level for both the aerogel skeleton and quartz fibers, the stress–strain curves exhibit an upward trend, marking the end of the yield stage and transition into the densification stage (35–60% strain). During this stage, the compressed aerogel network and buckled fibers undergo further compaction, causing stress to rise sharply with increasing strain. CT slices at 60% (Figure 6(e_8_)) strain show more severe buckling of the Z-direction fibers, with increased interfacial failure between fibers and the aerogel matrix. In summary, the structural changes within the aerogel composites under Z-direction compression indicate that fiber buckling and the resulting interfacial debonding are primary failure mechanisms during material loading.

Under XY-direction compression, stress increases linearly with strain initially. The linear elasticity persists until a strain of approximately 6–13%, where the stress reaches the elastic limit. Subsequently, the composites enter the yield stage, where stress fluctuates around a local range as strain increases, indicating that NQF/PR composites gradually undergo permanent structural damage. When strain further increases beyond 35%, stress begins to rise rapidly again, with the rate of increase gradually accelerating. During this stage, the composites are progressively compressed, accompanied by structural failures such as macroscopic delamination, leading to complete material failure.

Due to the anisotropic structure of needled quartz fiber felt, the compressive behavior of NQF/PR composites differs between the Z-direction and the XY-direction. In the Z-direction, no distinct yield point is observed. However, when strain exceeds approximately 10%, the slope of the stress–strain curve decreases. Therefore, the compressive strength σ_10%_ in the Z-direction is defined as the stress at 10% strain. The elastic modulus in the Z-direction is determined from the slope of the stress–strain fitting line for strains ranging from 0% to 10%. For the XY-direction, compression exhibits maximum stress before entering the yield stage. Therefore, the compressive strength σ_max_ is defined as the maximum stress in the linear elastic region, and the elastic modulus is determined from the slope of the stress–strain fitting line in the linear elastic stage. Based on these definitions, the compressive strength and compressive modulus of the composites were analyzed. The calculation results are shown in Figure 6b,d, and Table 5. Comparing the data in the figures and table reveals that both the compressive strength and compressive modulus of the NQF/PR increase with the phenolic resin concentration, both in the Z- and XY-directions. In the Z-direction, compressive strength and compressive modulus increase from 0.237 MPa and 1.465 MPa for NQF/PR-10 to 7.447 MPa and 79.299 MPa for NQF/PR-30, respectively. Similarly, in the XY-direction, they increase from 0.379 MPa and 2.279 MPa to 14.913 MPa and 175.639 MPa. This trend is primarily attributable to two factors. Firstly, as the phenolic resin concentration in the precursor solution increases, the aerogel matrix encapsulates quartz fibers more tightly. The structure not only provides stronger lateral support to the fibers, effectively limiting their elastic rotation and bending, but also enhances load transfer efficiency by strengthening interfacial bonding. On the other hand, the increased phenolic resin concentration directly elevates the solid-phase content within the aerogel matrix, resulting in a denser microstructure. Structural densification fundamentally enhances the inherent load-bearing capacity of the matrix.

Comparing the compressive properties of the same NQF/PR in the Z-direction and XY-direction reveals that both compressive strength and compressive modulus in the Z-direction are lower than their counterparts in the XY-direction. The varying compression properties in different directions are primarily attributed to the anisotropic fiber arrangement of the needled quartz fiber felt. The needled quartz fiber felt exhibits anisotropic distribution in the in-plane direction, while adopting a layered arrangement in the thickness direction. Quartz fibers within each layer are intertwined through the action of the needles. When loaded in the XY-direction, the load direction is parallel to the distribution plane of most fibers. A large number of randomly oriented fibers can participate in load bearing, resulting in high compressive strength and modulus at the macroscopic level. Conversely, when loaded in the Z-direction, the majority of fibers are oriented perpendicularly to the compressive stress direction, with the limited number of needled fibers aligned along the stress direction. Consequently, under Z-direction loading, only the needled fibers and the phenolic aerogel structure effectively bear the load, which results in the composites exhibiting weaker compressive strength. The mechanical properties of the NQF/PR in this study are higher than those of foam and rock wool reported in the literature [28,29,30,31], providing a solid foundation for thermal insulation applications.

This study used X-ray micro-CT to reveal the structural evolution of fiber buckling and interfacial debonding during loading in NQF/PR composites, providing microscopic structural evidence for understanding their macroscopic mechanical behavior. The prepared NQF/PR materials exhibited a maximum compressive strength of 7.447 MPa in the Z-direction and a maximum compressive modulus of 79.299 MPa. In the XY-direction, the maximum compressive strength reached 14.913 MPa, with a maximum compressive modulus of 175.639 MPa. The disparity in compressive strength and behavior between the Z-direction and XY-direction primarily stems from the anisotropic properties of the needled quartz fiber felt.

### 3.4. Thermal Insulation Properties and Mechanism

**Figure 7:**
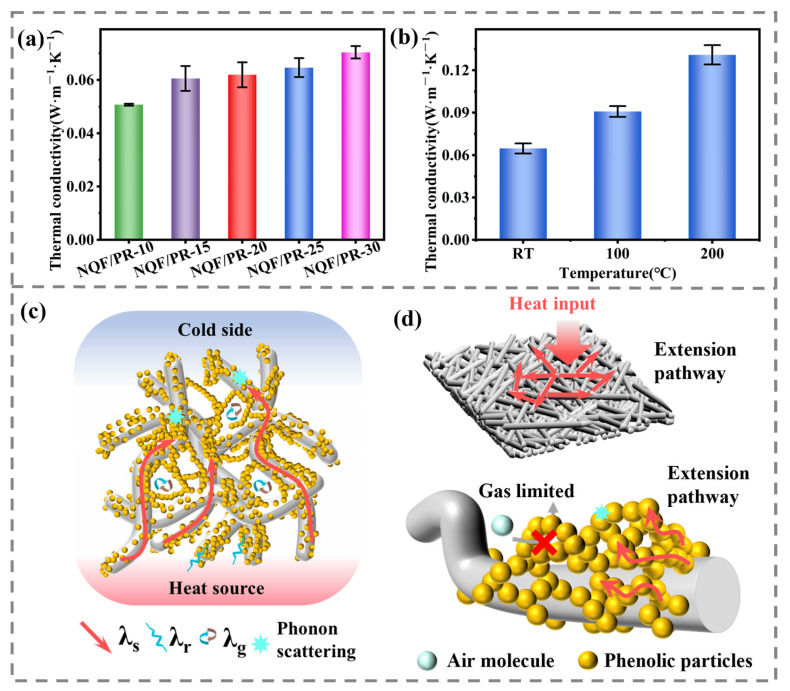
(**a**) Thermal conductivity of NQF/PR at room temperature. (**b**) Thermal conductivity of NQF/PR-25 at various temperatures. (**c**) Heat transfer mechanism of NQF/PR. (**d**) Thermal insulation mechanism of needled quartz fiber felt and nano-phenolic aerogel.

Thermal conductivity is a key indicator for evaluating the thermal insulation performance of porous materials and is closely related to the microstructure of materials. In this study, the thermal conductivity of NQF/PR aerogel composites at room temperature was tested, with the results shown in Figure 7a. Specifically, the thermal conductivities of NQF/PR-10 to NQF/PR-30 were 0.0507, 0.0605, 0.0619, 0.0646, and 0.0704 W·m^−1^·K^−1^, respectively. All values were relatively low, indicating that NQF/PR composites exhibit excellent thermal insulation properties. Generally, higher material density correlates with higher thermal conductivity. However, in this study, NQF/PR-30 with a density of 0.484 g/cm^3^ exhibited a significantly lower thermal conductivity than the lightweight needled carbon fiber felt/phenolic resin aerogel composite with a density of 0.37 g/cm^3^ [20], being only 39.11% of the latter.

The heat transfer mechanism of NQF/PR is shown in Figure 7c. Traditionally, materials primarily transfer heat through three pathways: radiation, convection, and conduction. Conduction includes both gas and solid conduction, which can be specifically described by Equation (3) [32,33].

(3) $$\lambda_{total}=\lambda_{r}+\lambda_{c}+\lambda_{g}+\lambda_{s}$$

where *λ_total_* is the total thermal conductivity, *λ_r_*, *λ_c_*, *λ_g_*, and *λ_s_* represent the thermal conductivities contributed by thermal radiation, convection, the gas, and the solid, respectively.

Typically, when the pore size is less than 2 mm, gas convection is suppressed, and the effect of convective heat transfer is typically negligible [34,35]. In NQF/PR, pore sizes mainly range from nanometers to micrometers, so *λ_c_* can be neglected. Furthermore, at room temperature, the contribution of *λ_r_* is generally minor [36]. Consequently, heat transfer in NQF/PR at room temperature primarily depends on *λ_g_* and *λ_s_*.

Considering the Knudsen effect, the *λ_g_* can be calculated using Equations (4) and (5) [37].

(4) $$\lambda_{g}=\xi \frac{\lambda_{g}^{0}}{1+2\alpha K_{n}}$$

(5) $$K_{n}=l_{g}/D$$

where *ξ* is the porosity, $\lambda_{g}^{0}$ is the thermal conductivity of the gas ($\lambda_{g}^{0}$ = 0.026 W·m^−1^·K^−1^ for air at room temperature and pressure) [34,35], *α* is a parameter taking into account energy transfer efficiency between gas molecules and the solid interface (*α* ≈ 2 for air) [37,38], *K_n_* is the Knudsen number, *l_g_* is the mean free path of gas molecules (70 nm for air at room temperature) [39], and *D* is the pore diameter within the material.

Substituting the relevant parameter values into Equations (4) and (5), *λ_g_* was calculated. Subsequently, *λ_s_* was determined based on the measured *λ_total_*. The final calculation results are listed in Table 6.

As shown by the quantitative analysis in Table 6, with increasing phenolic resin concentration in the aerogel precursor solution, the gas thermal conductivity of the NQF/PR aerogel composites gradually decreases. The contribution rate of gas thermal conductivity decreases from 19.01% to 3.76%, while the solid thermal conductivity progressively increases, with the contribution rate rising from 80.99% to 96.24%. This change is primarily attributed to microstructural evolution. As the phenolic resin concentration increases, a denser gel network forms within the material, resulting in progressively smaller average pore sizes and a gradual decrease in porosity. Consequently, the thermal motion of gas molecules becomes restricted (Figure 7d) [40].

Although the dense network structure enhances solid thermal conductivity, the thermal conductivity of NQF/PR remains lower than that of carbon foam (0.095–0.2 W·m^−1^·K^−1^) [41,42] and low-density phenolic resin matrix composites (0.129–0.298 W·m^−1^·K^−1^) [43], primarily due to elongated heat transfer paths and phonon scattering at solid–solid and solid–gas interfaces. As shown in Figure 7d, randomly distributed fibers within the XY plane force heat flow to follow tortuous paths, significantly extending the thermal transmission path [44]. The uniformly distributed phenolic aerogel within the fiber felt enhances phonon scattering at the solid–solid and solid–gas interfaces, increasing interfacial thermal resistance. Moreover, it further complicates and randomizes the thermal conduction pathways, thereby inhibiting efficient heat transfer.

The above calculations employ the average pore diameter D. In reality, the prepared NQF/PR composites exhibit a multiscale pore structure comprising micropores, mesopores, and macropores. Micropores possess diameters smaller than the mean free path of molecules in air, severely restricting heat transfer via molecular kinetic energy exchange. In macropores, gas molecules behave nearly as free gas. To analyze the uncertainty introduced by using the average pore diameter, we adjusted the pore diameter values to 0.8–1.2 times the average pore diameter. The solid conduction contribution calculated for NQF/PR-10 ranged from 79.02% to 83.34%, approaching 80%. For a constant energy transfer coefficient *α*, according to Equation (4), *λ_g_* is inversely proportional to *α*. When *α* is reduced to 1.63 [45], the solid conduction contribution calculated by NQF/PR-10 remains at 78.78%, approaching 80%.

As shown in Figure 7b, this study tested the thermal conductivities of sample NQF/PR-25 at 100 °C and 200 °C, which were 0.0908 and 0.131 W·m^−1^·K^−1^, respectively. Compared to the thermal conductivity at room temperature, the thermal conductivity of the sample exhibited an increasing trend with rising temperature. The change primarily resulted from the enhanced contribution of *λ_r_*. The radiative thermal conductivity *λ_r_* can be calculated using the Rosseland Equation [46]:

(6) $$\lambda_{r}=\frac{16\sigma n^{2}T^{3}}{3\rho e^{*}}$$

where *σ* is the Stefan–Boltzmann constant, *n* is the refractive index, *T* is the absolute temperature, *ρ* is the material density, and e* is the extinction coefficient.

The Rosseland Equation (6) demonstrates that the radiative thermal conductivity exhibits a strong positive correlation with temperature, causing radiative heat transfer to become increasingly significant as temperature rises.

**Figure 8:**
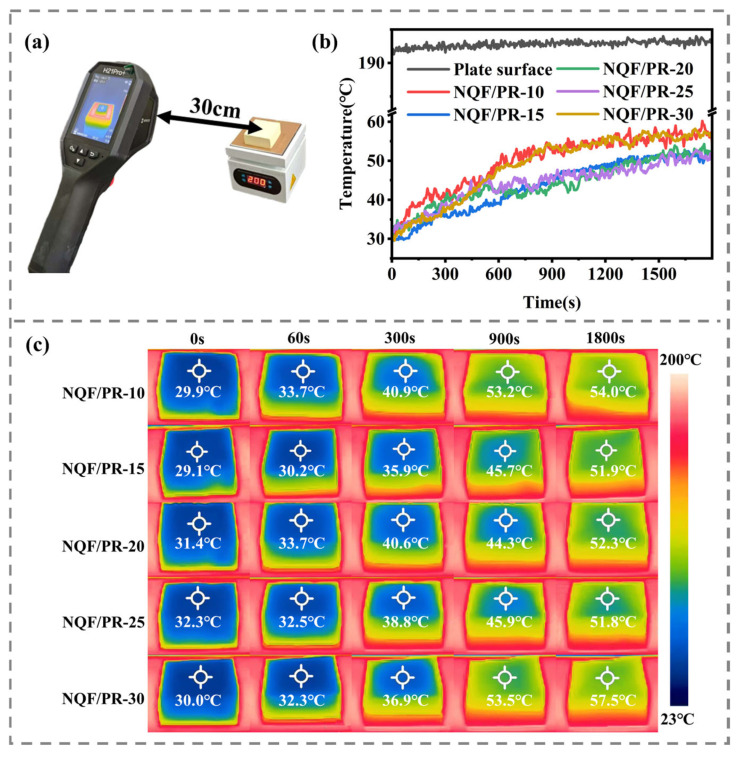
Thermal insulation testing of 20 mm thick NQF/PR on a 200 °C heating plate. (**a**) Test schematic. (**b**) Backside temperature curves of samples. (**c**) Infrared thermal images at different times.

To further evaluate the thermal insulation performance of NQF/PR aerogel composites, samples with thicknesses of 20 mm were placed on a 200 °C heating plate for constant temperature testing. An infrared thermal imager was used to record the infrared thermal distribution and backside temperature changes. The test setup is shown in Figure 8a. Temperature readings were recorded every 5 s over the 1800 s test duration. The backside temperature curves and infrared thermal images for samples are presented in Figure 8b,c. All NQF/PR samples exhibited similar thermal responses. When placed on a 200 °C heating plate, the back temperature slowly increased until thermal equilibrium was reached. At 1800 s, the back temperatures of the NQF/PR samples were 54.0, 51.9, 52.3, 51.8, and 57.5 °C respectively, representing increases of 24.1, 22.8, 20.9, 19.5, and 27.5 °C compared to the initial temperatures. The temperature difference between the sample backside and the heat source is approximately 140 °C, confirming the excellent thermal insulation properties of the prepared NQF/PR composites.

**Figure 9:**
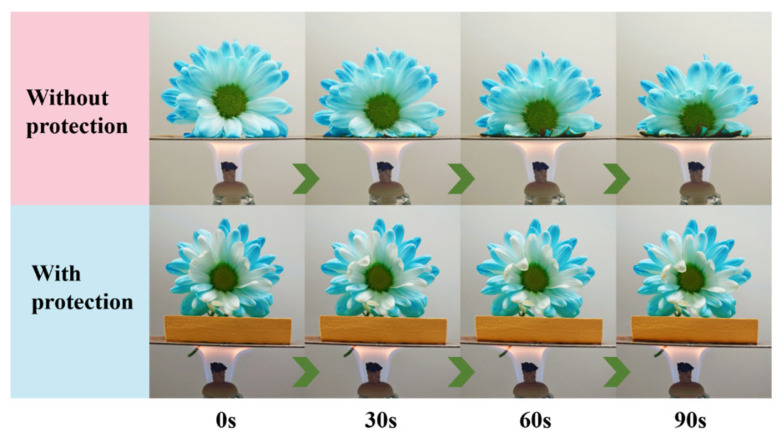
Thermal protection of flowers placed on an aluminum plate using a 10 mm thick NQF/PR-25.

Additionally, a flower placed on an aluminum plate covered with a 10 mm thick NQF/PR-25 sample was continuously heated from below with an alcohol burner flame for 90 s. As shown in Figure 9, the flower directly on the aluminum plate was quickly scorched, while the flower protected by the NQF/PR-25 sample exhibited only slight wilting. The results demonstrate that the NQF/PR composites effectively insulate heat, providing superior thermal protection for objects on the backside.

**Figure 10:**
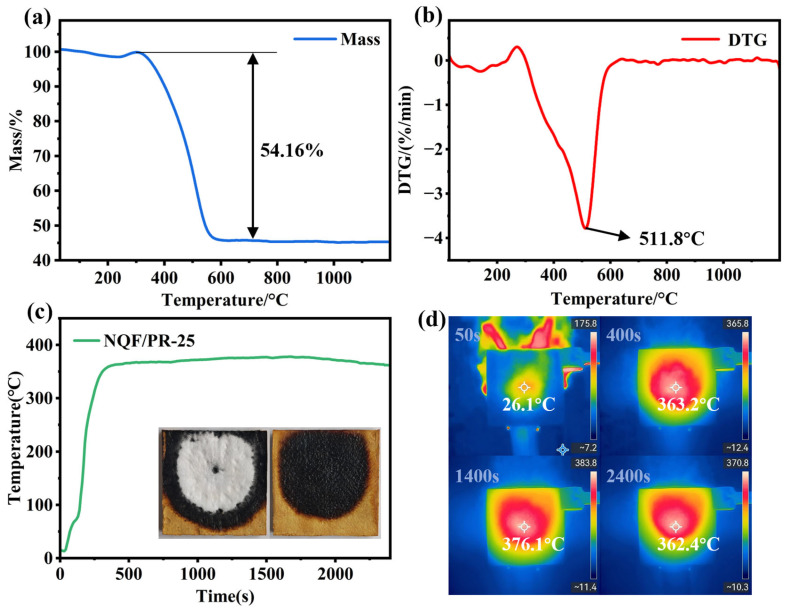
(**a**) TG curve of NQF/PR-25. (**b**) DTG curve of NQF/PR-25. (**c**) Back surface temperature curve of NQF/PR-25 tested for 2400 s under a butane torch flame at temperatures up to 1300 °C. (**d**) Infrared thermal images at different time points during the butane torch flame test.

The thermal stability of NQF/PR-25 was analyzed in air, as shown in Figure 10a,b. Between room temperature and 300 °C, a slight mass loss occurred, primarily due to the volatilization of water absorbed within the aerogel pore structure. Between 300 °C and 600 °C, NQF/PR-25 exhibited significant weight loss, losing approximately 54.16% of its mass. This stage primarily involved the breakdown and intense pyrolysis of the phenolic resin organic framework, yielding small molecules such as CO, CO_2_, and H_2_O. Mass loss was substantially driven by gas evolution at high temperatures. Between 600 °C and 1200 °C, the mass of NQF/PR-25 remained nearly constant, as indicated by the flattening of the TG curve. This indicated that the phenolic aerogel had undergone near-complete pyrolysis. The residue exhibited excellent thermal stability within this temperature range, showing no further breakdown or oxidation reactions.

The thermal insulation performance of the 10 mm thick NQF/PR-25 sample at high temperatures was evaluated using a butane torch flame with a maximum operating temperature of 1300 °C. The backside temperature and infrared thermal images of the NQF/PR-25 sample were recorded, as shown in Figure 10c,d. At the start of the test, the temperature on the backside of the sample rose slowly, reaching approximately 360 °C after 400 s. It then remained nearly stable, indicating that the NQF/PR-25 aerogel composite exhibits good thermal insulation properties at high temperatures. As phenolic aerogel pyrolyzes at high temperatures, the generated gases carry away heat, thereby protecting the internal material. Concurrently, the smaller pore size and denser aerogel framework within NQF/PR-25 effectively prevent heat and gas injection into the material, thereby reducing thermal erosion during ablation.

Table 7 quantitatively compares the compressive properties and thermal conductivity of the NQF/PR-25 prepared in this study with those of previously reported fiber-reinforced phenolic aerogel composites. It can be observed that the quartz fiber-reinforced phenolic resin composites reported by Tao et al. [47], although having densities close to NQF/PR-25, exhibit significantly inferior thermal conductivity and compressive properties compared to the NQF/PR-25 composite. Particularly in the Z-direction, the compressive strength of NQF/PR-25 exceeds the quartz fiber-reinforced phenolic resin composites by more than threefold. The needled quartz/carbon fiber preform reinforced phenolic aerogel composite prepared by Gu et al. [48] exhibits a thermal conductivity close to that of NQF/PR-25, but its compressive strength in the Z-direction is significantly inferior to those of the NQF/PR-25 composite. Overall, NQF/PR-25 exhibits an excellent combination of low thermal conductivity and superior compressive mechanical properties at a relatively low density.

## 4. Conclusions

This study prepared phenolic aerogel composites reinforced with needled quartz fiber felt using vacuum impregnation, sol–gel, and ambient pressure drying. By controlling the phenolic resin concentration in the precursor solution, the pore structure of aerogel was regulated. Among the prepared samples, the NQF/PR-25 aerogel composite achieves a favorable balance between thermal insulation and mechanical properties, exhibiting both a low thermal conductivity and high compressive strength. X-ray micro-CT revealed the buckling process of Z-direction fibers under compressive loading and the evolution of fiber–matrix interfaces. Furthermore, by combining theory with experiment, the internal heat transfer mechanism was clarified, revealing that solid conduction constitutes the primary contributor (>80%) to the overall thermal conductivity at room temperature. This study provides a methodological reference for optimizing the properties of fiber-reinforced aerogel composites.

## Figures and Tables

**Table 1:** Component ratios of phenolic aerogel precursor solutions.

Samples	HMTA (g)	H_2_O (g)	TsOH (g)	TPPR (g)	n-Butanol (g)
PR-10	1.72	1.03	0.46	11.47	100
PR-15	2.78	1.67	0.74	18.56	100
PR-20	4.03	2.42	1.08	26.88	100
PR-25	5.51	3.31	1.47	36.76	100
PR-30	7.32	4.39	1.95	48.78	100

**Table 2:** Dimensions of NQF/PR aerogel composites before and after drying.

Samples	Before Drying			After Drying		
	Length /mm	Width /mm	Height /mm	Length /mm	Width /mm	Height /mm
NQF/PR-10	102.09	101.02	11.75	102.04	100.91	11.74
NQF/PR-15	101.80	101.47	12.15	101.79	101.45	12.13
NQF/PR-20	101.73	102.26	12.74	101.71	102.23	12.70
NQF/PR-25	102.41	102.29	12.19	102.38	102.27	12.15
NQF/PR-30	101.84	100.98	11.62	101.65	100.80	11.61

**Table 3:** Density and porosity of NQF/PR aerogel composites.

Samples	NQF/PR-10	NQF/PR-15	NQF/PR-20	NQF/PR-25	NQF/PR-30
Density (g/cm^3^)	0.263	0.305	0.351	0.406	0.484
Porosity (%)	84.94	81.36	77.86	73.41	67.69

**Table 4:** Pore structure parameters of NQF/PR aerogel composites.

Samples	S_BET_ (m^2^/g)	S_micro_ (m^2^/g)	V_total_ (cm^3^/g)	V_micro_ (cm^3^/g)
NQF/PR-10	13.521	1.366	0.058	0.00034
NQF/PR-15	29.035	4.920	0.124	0.00186
NQF/PR-20	52.829	9.772	0.226	0.00388
NQF/PR-25	78.690	11.249	0.474	0.00420
NQF/PR-30	82.311	12.406	0.466	0.00417

Note: S_BET_: BET surface area; S_micro_: Micropore surface area; V_total_: Total pore volume calculated at a relative pressure of 0.99; V_micro_: Micropore volume.

**Table 5:** Mechanical properties of NQF/PR aerogel composites.

Samples	Z-Direction		XY-Direction	
	Strength (MPa)	Modulus (MPa)	Strength (MPa)	Modulus (MPa)
NQF/PR-10	0.237 ± 0.015	1.465 ± 0.141	0.379 ± 0.060	2.279 ± 0.341
NQF/PR-15	0.734 ± 0.051	5.890 ± 0.601	1.807 ± 0.173	34.426 ± 4.875
NQF/PR-20	1.877 ± 0.055	16.387 ± 0.870	4.543 ± 0.170	89.649 ± 12.856
NQF/PR-25	3.873 ± 0.065	35.904 ± 0.920	7.702 ± 0.456	153.911 ± 19.065
NQF/PR-30	7.447 ± 0.095	79.299 ± 2.719	14.913 ± 0.533	175.639 ± 17.726

**Table 6:** Gas and solid conductivity of NQF/PR aerogel composites at room temperature.

Samples	*λ_g_* W·m^−1^·K^−1^	*λ_s_* W·m^−1^·K^−1^	Fraction of the Gas Conduction (%)	Fraction of the Solid Conduction (%)
NQF/PR-10	0.00964	0.0411	19.01	80.99
NQF/PR-15	0.00868	0.0518	14.34	85.66
NQF/PR-20	0.00691	0.0550	11.16	88.84
NQF/PR-25	0.00340	0.0612	5.26	94.74
NQF/PR-30	0.00265	0.0678	3.76	96.24

**Table 7:** Properties of fiber-reinforced phenolic aerogel composites.

Materials	Density (g/cm^3^)	Thermal Conductivity at RT (W·m^−1^·K^−1^)	Compressive Strength in the Z-Direction (MPa)	Compressive Strength in the XY-Direction (MPa)	Ref.
NQF/PR-25 in this work	0.406	0.0646	3.873	7.702	
NQF/PR	0.372–0.398	0.086–0.094	0.67–1.04	4.03–5.08	[47]
NQCF/PR	0.23	0.066	0.27		[48]
NQF/PR	0.3		2.16	3.76	[49]
NQF/PR	0.31–0.36	0.017–0.031	0.43–0.76	1.76–2.84	[50]

## Data Availability

The original contributions presented in this study are included in the article. Further inquiries can be directed to the corresponding author.
